# Physical performance and glycemic control under SGLT-2-inhibitors in patients with type 2 diabetes and established atherosclerotic cardiovascular diseases or high cardiovascular risk (PUSH): Design of a 4-week prospective observational study

**DOI:** 10.3389/fcvm.2022.907385

**Published:** 2022-07-22

**Authors:** Devine S. Frundi, Eva Kettig, Lena Luise Popp, Melanie Hoffman, Marine Dumartin, Magali Hughes, Edgar Lamy, Yvonne Joko Walburga Fru, Arjola Bano, Taulant Muka, Matthias Wilhelm

**Affiliations:** ^1^Berner Klinik Montana, Zentrum für Medizinische und Neurologische Rehabilitation, Crans-Montana, Switzerland; ^2^Permanence Médicale, Hôpital de Sierre, Sierre, Switzerland; ^3^Nuffield Department of Population Health (NDPH), University of Oxford, Oxford, United Kingdom; ^4^Institute of Social and Preventive Medicine (ISPM), University of Bern, Bern, Switzerland; ^5^Center for Preventive Cardiology, University Hospital Bern and University of Bern, Bern, Switzerland

**Keywords:** SGLT-2-inhibitors, atherosclerotic cardiovascular disease, high cardiovascular risk, type II diabetes mellitus, glycemic control, physical performance

## Abstract

**Background:**

Type 2 diabetes (T2D) is associated with limitation in physical performance. Results from animal studies report enhancement of physical performance in T2D rodents treated with sodium glucose cotransporter 2 inhibitors (SGLT2is). However, in human patients with T2D and established atherosclerotic cardiovascular disease (ASCVD) or high cardiovascular risk, the impact of guideline directed SGLT2i medication on physical performance has not been sufficiently examined.

**Objectives:**

The main objectives of this study are thus firstly, to assess the changes in physical performance after 4 weeks of exercise therapy in patients with established ASCVD or high cardiovascular risk categorized into three groups according to their glycemic control at baseline. Secondly, to investigate the association of glycemic control at baseline and new guideline directed antidiabetic treatment (inadequate glycemic control and diabetes + new SGLT2i vs. adequate glycemic control and diabetes vs. no diabetes) with change in physical performance.

**Methods and design:**

This is a 4-week prospective observational study of 450 participants with established ASCVD or high cardiovascular risk with or without T2D and without previous SGLT2i medication undergoing exercise therapy during inpatient rehabilitation in a single center in Switzerland. Upon admission, participants are categorized into 3 groups of 150 participants each according to their glycemic control. Group I consisting of participants with inadequately controlled T2D defined as mean fasting plasma glucose (FPG) of ≥7 mmol/L, who are consequently administered new treatment with an SGLT2i. Group II comprises of participants with adequately controlled T2D with mean FPG of <7 mmol/L requiring no antidiabetic medication change. Group III consists of participants with no diabetes and mean FPG of ≤ 5.5 mmol/L. Primary outcomes are 6-min walk distance and rate of perceived exertion. Secondary outcomes are echocardiographic parameters (left ventricular mass index; global longitudinal strain average; end-diastolic volume), fatigue, muscle, metabolic, and anthropometric measures.

**Ethics and dissemination:**

This study is conducted in accordance with the Declaration of Helsinki with ethical approval from the Cantonal Ethical Commission of Bern, Switzerland. The results will be published in a peer-reviewed journal. The implementation and reporting will be according to the SPIRIT guidelines.

**Study protocol registration:**

https://www.clinicaltrials.gov/, identifier: NCT03422263.

## Introduction

Type 2 diabetes (T2D) has been associated with limitation in physical performance in individuals with or without atherosclerotic cardiovascular diseases (ASCVD) (1–4). These impairments in physical performance, measured as submaximal or maximal exercise capacity, manifest even with adequate glycemic control and in the absence of overt signs or symptoms of established ASCVD (1, 4–6). This is of clinical relevance because low physical performance in addition to poor glycemic control strongly predicts long-term cardiovascular mortality in individuals with T2D (7–11).

Accordingly, improvement of exercise capacity is considered an important prevention and therapeutic target in existing guidelines to alleviate the risk of atherosclerotic cardiovascular events and mortality in individuals with T2D and/or ASCVD (12–15). Furthermore, because independent of diabetes, fasting plasma glucose is associated with risk of ASCVD, the guidelines also highlight the need for adequate glycemic control (11, 15). Consequently, algorithms for the use of medical therapy have been developed in recent years for the treatment of T2D based on individualized cardiovascular risk profiles with sodium glucose cotransporter 2 inhibitors (SGLT2is) being recommended mainly for individuals with congestive heart failure, established ASCVD or high cardiovascular risk (16–19). These recommendations are based on the results of major cardiovascular outcome trials on SGLT2is, namely empagliflozin, dapagliflozin, and canagliflozin, in patients with T2D which showed that this drug class consistently reduces the risks of heart failure hospitalisations, progression of renal disease as well as moderately reduces the risk of major adverse cardiovascular events, or at least some features of them (20–23). Data suggest these benefits are independent of glycemic control and can be apparent early upon initiation of treatment (24). However, the precise mechanisms of these benefits remain unclear.

Based on recent studies in animals, a possible mechanism can be the enhancement of exercise capacity after 4 weeks of SGLT2i treatment in murine models of T2D and heart failure. The results showed that lowered exercise capacity was restored through central and peripheral mechanisms by improvement of myocardial function and activation of skeletal muscle fatty acid oxidation (25, 26). In humans, although a few studies with small sample sizes suggested that empagliflozin was associated with early improvement in exercise capacity in T2D patients with heart failure (27, 28), others could not replicate these findings on empagliflozin and canagliflozin (29, 30). In a randomized, double blinded study of exercise therapy and dapagliflozin vs. exercise therapy and placebo, dapagliflozin treatment resulted in higher increase in peak exercise capacity from baseline when compared with exercise therapy and placebo despite elevated fasting plasma glucose levels and abrogated increased insulin sensitivity when compared to exercise therapy and placebo (31). These findings suggest that the influence of dapagliflozin on physical performance measured as peak exercise capacity may possibly be independent of glycemic control and that when combined with exercise therapy as recommended by guidelines, dapagliflozin may even contribute to a poorer glycemic control.

To the best of our knowledge, the above findings have not been examined patients with T2D and established ASCVD or high cardiovascular risk, as such, in patients where guideline recommendations strongly favor both improvement of physical performance and first-line medical therapy with an SGLT2i (15, 19).

Given this background, the main aims of this study are firstly, to investigate the changes in physical performance and glycemic control 4 weeks after initiation of guideline-directed medical therapy of an SGLT2i and exercise therapy in patients with established ASCVD or high cardiovascular risk and T2D with initial inadequate glycemic control; secondly, to investigate the changes in physical performance and glycemic control 4 weeks after exercise therapy only in patients with established ASCVD or high cardiovascular risk and T2D with adequate glycemic control; thirdly, to investigate the changes in physical performance 4 weeks after exercise therapy only in non-diabetic patients with established ASCVD or high cardiovascular risk; and fourthly to investigate the cross-sectional and longitudinal association of glycemic control at baseline and new guideline directed antidiabetic treatment (inadequate glycemic control and diabetes + new SGLT2i vs adequate glycemic control and diabetes vs. no diabetes) with physical performance and other outcomes influencing physical performance (secondary outcomes), in patients with established ASCVD or high cardiovascular risk after adjusting for baseline covariates.

## Methods and analysis

### Study design

**Figure 1:**
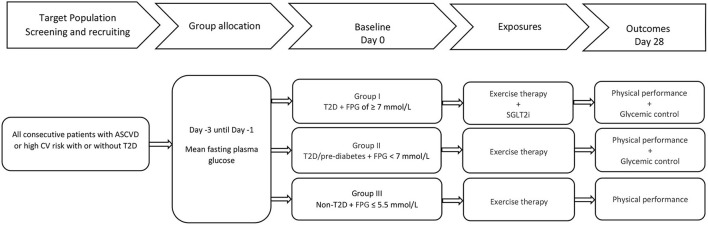
Flowchart of the PUSH study. ASCVD, atherosclerotic cardiovascular diseases; CV, cardiovascular; T2D, type II diabetes mellitus; FPG, fasting plasma glucose; SGLT2i, sodium glucose cotransporter 2 inhibitor.

The PUSH is a real-world, 4 week prospective, three-arm observational study of patients with established ASCVD or high cardiovascular risk with and without T2D undergoing regular exercise therapy during inpatient rehabilitation in a single center in Switzerland. Employing a multi-group design and a two-step analytic approach, the study investigates the changes in physical performance (and glycemic control) in 3 categories of glycemic control after 4 weeks. Furthermore, the associations between the 3 categories of glycemic control and new guideline directed antidiabetic treatment (inadequate glycemic control and diabetes + new SGLT2i vs. adequate glycemic control and diabetes vs. no diabetes) with physical performance are evaluated in cross-sectional and prospective analyses. A flow chart of the study is shown in Figure 1.

#### Eligibility criteria

All consecutive patients with established ASCVD or high cardiovascular risk with or without T2D who met the inclusion criteria summarized below were prospectively enrolled as from 1 January 2018 and data collection lasted until 30 September 2020. Individuals below 40 years of age upon admission were excluded due to the low incidence of ASCVD in people under 40 years (32). For participants with T2D, T2D was determined by self-report with verification of medical records, current treatment or FPG ≥ 7 mmol/L and symptoms of hyperglycemia with random plasma glucose ≥ 11.1 mmol/L (33). Individuals who had a clinical history strongly suggestive of type I diabetes were excluded.

#### Inclusion criteria

1. Ambulatory patients, defined as able to walk about and not bedridden, with or without walking aids admitted following a medical or surgical condition warranting inpatient rehabilitation,
2. willing to give informed written consent,
3. with established ASCVD or high cardiovascular risk with and without T2D were included. ASCVD and high cardiovascular risk were defined as follows:

a. established ASCVD: coronary artery disease (CAD), cerebrovascular disease (CeVD), peripheral artery disease (PAD) and ischemic congestive heart failure (CHF),
b. high cardiovascular risk: as defined in the 2016 European Guidelines on cardiovascular disease prevention in clinical practice (13). consisting of patients without clinically manifest ASCVD with markedly elevated single cardiovascular risk factors (hypertension with blood pressure ≥180/110 mmHG, blood lipid profile with cholesterol > 8 mmol/L, metabolic syndrome and central obesity with body mass index ≥40 kg/m^2^ in patients below 60 years of age), or with T2D plus any additional risk factor, or with moderate chronic kidney disease defined as glomerular filtration rate between 30 and 59 mL/min/1.73 m^2^, or with a calculated systematic coronary risk estimation (SCORE) ≥5 and <10 % (13), or with scores above the high risk cut-off using the American College of Cardiology (ACC)/American Heart Association (AHA) score of ≥20% (34, 35).

#### Exclusion criteria

1. Patients who were not willing to give informed consent or declined suggestions of guideline directed medical therapy,
2. who were not able to walk,
3. in lower cardiovascular risk categories,
4. had a history of unstable angina or acute myocardial infarction in the 4 weeks prior to baseline or any terminal cardiovascular, respiratory, musculoskeletal, or neurological condition that relevantly limited patient's physical performance and/or movement defined as 6-min walk distance of <25 m at baseline,
5. had relevant pain limiting physical performance and/or movement defined as pain numeric rating scale (NRS) of above 5/10 at rest,
6. had an active malignancy,
7. had advanced renal disease defined as glomerular filtration rate (GFR) permanently below 30 mL/min/1.73 m^2^ in the 12 weeks prior to admission,
8. were already under treatment with SGLT2i upon admission were excluded. Participants were considered already under treatment with an SGLT2i if they received continuous SGLT2i medication for longer than 3 days prior to admission.

### Study group categories

Glycemic control was assessed within the first 3 days upon admission by measurement of fasting plasma glucose (FPG) with the widely used diagnostic cut-off of ≥7 mmol/L employed to define inadequate glycemic control (33, 36). Hemoglobin A_1c_ (HBA_1c_) testing was equally done in all participants upon admission but was not used to define inadequate glycemic control in this cohort to circumvent the bias linked to participants with conditions that affect erythrocyte turnover such as acute bleeding and/or erythrocyte transfusion during the acute hospitalization prior to rehabilitation (33). HbA_1c_ was however used as one of the parameters to distinguish participants with diabetes or impaired glucose metabolism from those with normal glucose metabolism as described below.

Depending on their glycemic control upon admission and following guideline recommendations, participants were categorized to one of three groups using clinically defined cut-off categories of FPG levels as follows (11):

Group I: Participants with inadequately controlled T2D defined as mean FPG of ≥7 mmol/l within the first 3 days upon admission under other standard of care medication, who were afterwards administered *de novo* SGLT2i treatment; namely either canagliflozin 100 mg, or dapagliflozin 10 mg, or empagliflozin 10 mg once daily, with the choice of which of the SGLT2is being at the discretion of the individual treating physician,

Group II: Participants with adequately controlled T2D, previous diagnosis of T2D or use of antidiabetic medication, with mean FPG <7 mmol/L; and participants with impaired fasting glycemia (IFG) or pre-diabetes, defined as mean FPG of 5.6–6.9 mmol/L and HbA_1c_ levels of 5.7–6.4 % (15, 33), under other standard of care treatment and no SGLT2i,

Group III: Participants with no previous diagnosis of T2D or use of antidiabetic medication and normal glucose metabolism defined as FPG of ≤ 5.5 mmol/L under other standard of care treatment and no SGLT2i were included as a reference group as previously described in the meta-analysis of prospective studies on diabetes mellitus, FPG, and risk of vascular disease (11).

### Rehabilitation program

The research was conducted in a single center at an altitude of 1,600 m in the Bernese Swiss Alps in Crans-Montana, Switzerland. All participants were exposed to supervised exercise therapy as follows: eight to twelve 30-min-sessions per week of physical activity, of which 4–5 sessions were aerobic exercises in a pool, on a cycloergometer, treadmill or outdoor moderate intensity walking, 2–3 sessions of muscle strengthening through resistance and stretching exercises, 2–4 sessions of occupational activity, proprioceptive and range of motion exercises supervised by blinded certified therapists. Exercise intensity was determined according to the rate of perceived exertion measured using the Borg category ratio (CR-10) as previously described (37, 38). Intensity was increased progressively according to the patient's tolerance. In addition, 30-min sessions of auxiliary non-physical activity with a dietician, psychologist, acupuncturist, art therapist or social worker were planned as needed.

For the PUSH study, participant motivation and participation were measured based on the session reports of blinded therapist during weekly interdisciplinary meetings using the Pittsburgh rehabilitation participation scale as previously described (39).

### Outcome measures: Assessment, blinding, and rationale

The PUSH assessed objective and subjective outcomes of physical performance in addition to hemodynamic, cardiorespiratory, and cardiometabolic risk variables. Outcome assessments were done upon admission (baseline), facultatively at week 2 and at week 4 (Table 1). All participants, direct care providers and assessors were masked to the outcomes under investigation throughout the study. Whereas, participant and care providers were blinded by categorically withholding supplementary information on the glycemic control categories described above and exposures under investigation, main outcome assessors (therapists and laboratory technicians) were masked by complete prohibition of access to patient medication data as per previously established institutional policy.

**Table 1:** Assessment time plan.

**Assessment**	**Time points during study**		
	**Baseline** ^a^	**Week 2**	**Week 4**
Screening for inclusion and exclusion criteria	°		
Participant characteristics^b^	°		
Status of medications^c^	°	°	°
Anthropometric and clinical parameters^d^	°	°	°
Laboratory tests^e^	°	°	°
Rehabilitation outcomes^f^	°	°	°
Transthoracic echocardiography^g^	°		°
Sonographic assessment of liver parenchyma (NAFLD)^h^	°		°
Muscle sonography^i^	°	°	°
Monitoring for adverse events^j^	Throughout stay		

a *Baseline was defined as the date of eligibility confirmation and inclusion in the study. If no data were available at baseline, the most recent data from the hospitalization prior to admission were used*.

b *Included information on age, sex, medical history, complications and comorbidities, smoker status, coffee consumption.*

c *Included antidiabetic agents, antihypertensives, lipid-lowering drugs, thrombocyte aggregation inhibitors, therapeutic anticoagulation, use of proton pump inhibitors, uric acid lowering drugs, antidepressants, opiates, NSAIDs, neuropathic pain treatment, vitamin D, iron, calcium, and magnesium substitution.*

d *Included body weight, BMI, waist and hip circumferences, estimation of skeletal muscle mass in kg using the Baumgartner equation, mean systolic and diastolic blood pressures, mean fasting plasma glucose (for all groups at baseline, for group I and II at week 2 and 4), peak expiratory flow rate, state of nocturia, pain numeric rating scale [NRS] (range 1–10).*

e *Included hemoglobin, thrombocyte count, magnesium, ionized calcium, ferritin, transferrin saturation, albumin, lipid profile (total cholesterol in mmol/L, mean HDL-Cholesterol in mmol/L, mean LDL-cholesterol in mmol/L, mean triglyceride in mmol/L), uric acid, NT-proBNP, kidney and liver parameters, CRP, vitamin D, TSH levels. HbA_1c_ testing was routinely done only upon admission (baseline).*

f *Included 6-Minute Test Walk Distance (6-MWD), percent of predicted 6-MWD, Borg Rate of perceived exertion 1–10, saturation and heart rate after 6-Min walk test; functional independence measure (FIM), use of walking aids. Baseline and week 4 only: hand grip strength, state, and severity of general, physical (motor), and cognitive fatigue.*

g *Included left ventricular mass index (LVMI), left atrial volume index (LAVI), global longitudinal strain average (GLS avg), end-diastolic volume (LVEDV).*

h *Included measurement of the inferior vena cava (IVC) diameter at expiration and at maximum inspiration, collapsibility index of the IVC, assessment of sonography criteria of non-alcoholic fatty liver disease (NAFLD).*

i *Included measurement of the muscle thickness ulna for the estimation of skeletal body mass in kg using muscle sonography (calculated as Muscle thickness ulna in cm × body height in m × 4.89–9.15), assessment of the pennate angle in degrees and echogenicity of the vastus lateralis muscle in arbitrary units, quadriceps muscle thickness.*

j *Included active monitoring for dehydration, polyuria, nocturia, urogenital infections, (euglycemic) diabetic ketoacidosis*.

#### Primary outcomes

The primary outcomes were defined as

i). walk distance (6-MWD in m),

ii). percent of predicted 6-MWD,

iii). rate of perceived exertion [Borg category ratio (CR-10)]

at the end of 6-minute walk test (6MWT) performed by a blinded certified therapist using the protocol outlined by the American Thoracic Society (ATS) with participant permitted to use a walking aid if needed (40). In brief, the 6MWT was performed over a 100 m long straight course within an enclosed level corridor. Standardized instructions were given to patients prior to each 6MWT. Patients wearing comfortable clothes and shoes were asked to walk as quickly as they can for 6 min to cover as much ground as possible. They were told that they could slow down or rest if necessary. The starting and turnaround points were indicated with a bright mark on the floor corresponding to the beginning and the end of each 90-meter lap, respectively, with further marks shown at 5-meter intervals. Heart rate (HR) and oxygen saturation (SpO2) were recorded prior to and upon completion of the 6MWT using an oximeter and finger sensor (OxyTrue A^®^, Bluepoint MEDICAL GmbH & Co.KG, Germany). At the end of the test, the distance was measured, and rate of perceived exertion was rated by the patient using a Borg category ratio (CR-10) scale. Because 6-MWD is directly related to age, height and sex and as such may give misleading results when used in groups with different age, sex and BMI distributions, the percent of predicted 6-MWD was also determined as a means of standardizing the outcome using norm-referenced equations (41, 42). For each participant, predicted 6-MWD was calculated as: 6-MWD_pred_ = 218 + (5.14 × height−5.32 × age)–[1.80 × weight + (51.31 × sex)]; where male = 1 and female = 0 (43). Additionally, percent of predicted 6-MWD was computed by dividing each participant's actual 6-MWD by the predicted 6-MWD and multiplying by 100, using the model parameter values (weight) at the assessment time point.

Measuring physical performance based on a submaximal exercise test, the 6MWT, is a distinctive choice as a primary outcome in this field given that previous exploratory studies focused on Cardiopulmonary Exercise Testing (CPET) with the measurement of peak exercise capacity (peak VO_2_) for assessing maximal aerobic capacity (27–30).

The decision to employ submaximal exercise testing as the primary outcome was based on several factors. First, the 6MWT is an inexpensive, efficient, safe and a well-tolerated method of assessing the functional exercise capacity of patients with ASCVD. It is widely used to follow the natural history of various diseases, and for measuring the response to medical interventions (40, 44, 45). Second, functional exercise capacity and mobility are composite of several factors targeted in rehabilitation programs in patients with established ASCVD and high cardiovascular risk with the goal of attaining physical activity levels in the community (13, 46–48). The 6MWT has been shown to correlate closely with physical performance levels in the community (49, 50) and to be strongly associated to clinical change and functional capacity following rehabilitation (51–53). The test reproduces the limitations of physical performance when undergoing activities of daily living such as dyspnea, fatigue, pain and weakness, while maximal aerobic capacity testing mainly measures the reserve capacity (thought to be barely tapped during daily activities) limited by exertion (54, 55). Third, evidence shows that the 6MWT is not associated with a relevant learning effect when repeated testing is performed in individuals with ASCVD (51, 56, 57). Fourth, minimal clinically important difference has been defined for the 6-MWT and helps determine whether a given change in the 6-MWT represents a clinically meaningful difference (58–60). Fifth, previous studies show that in patients with established ASCVD, poor physical performance measured with the 6-MWT is an independent predictor of survival and predicts risk of hospitalization, all-cause mortality, cardiovascular disease mortality, and mobility loss (44, 61–64). Together, these aspects mean the results of the primary outcomes of the PUSH should be indeed translatable to similar cohorts of patients with established ASCVD and high cardiovascular risk with or without a T2D comorbidity.

Furthermore, the duration of follow-up of 28 days was mainly based on a previous pilot study participants with similar comorbidities that showed that the SGLT2-inhibitor empagliflozin was associated with 1-month improvement in exercise capacity in T2D patients with symptomatic CHF measured amongst others using the 6-MWD and that this beneficial effect was also found for other surrogates of severity including quality of life measures and markers associated with congestive heart failure as measured in some of the secondary outcomes below (28).

#### Secondary outcomes

Secondary outcomes included surrogate variables related to or influencing physical performance measured as follows:

##### Transthoracic echocardiography parameters at rest

Main echocardiographic parameters such as left ventricular mass index (LVMI) in kg/m^2^, diastolic function (E/e' average), left atrial volume index (LAVI) in ml/m^2^, global longitudinal strain average (GLS avg) in %, doppler techniques used to measure end-diastolic volume (LVEDV) in ml, resting stroke volume (SV) in ml, left ventricular ejection fraction (LVEF) with datasets registered and stored on an Echo PAC software (GE Medical Systems Glattbrugg, Switzerland) for analysis offline by an independent cardiologist unaware of the treatment allocation using previously described guidelines and protocols (65–67).

**Figure 2:**
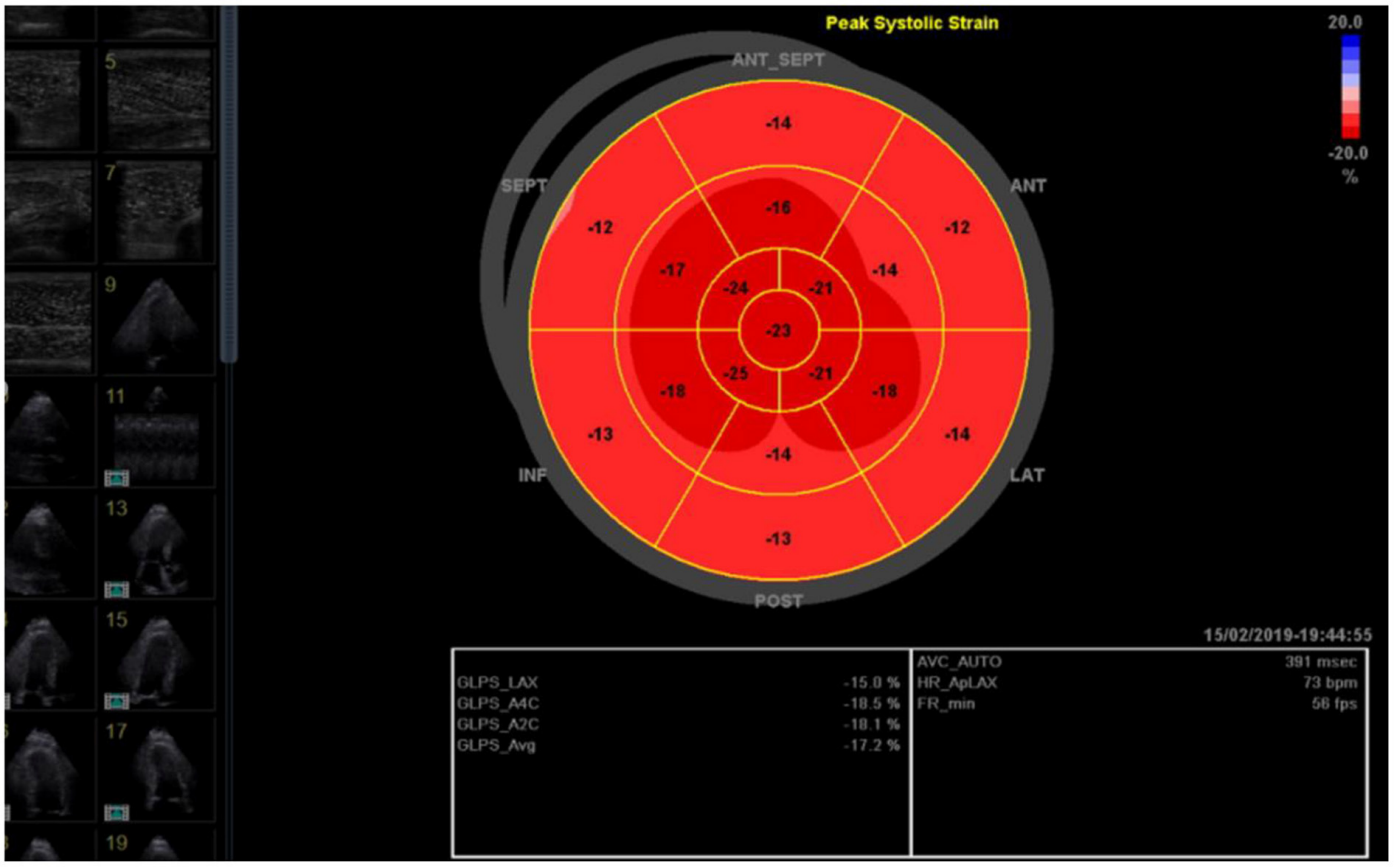
Global longitudinal strain average (GLS avg; here −17.2 %) as measured by a blinded cardiologist using images stored on an Echo PAC software.

It has been well-proven that physical performance correlates strongly with cardiac size (LV volumes, LV mass or whole heart volumes), both in athletic and non-athletic populations (68, 69). Likewise, there is a strong correlation between resting cardiac geometry measures (LV mass, LVEDV) and exercise capacity in individuals with T2D (6). And although these previous studies could not demonstrate a correlation between LVEF and exercise capacity in individuals, the PUSH is designed to explore early changes in newer echocardiographic measures of subclinical LV systolic dysfunction in the presence of a normal ejection fraction such as global longitudinal strain average [GLS avg] (Figure 2) (70).

##### Fatigue

Self-reported fatigue (continuous) and grade of fatigue severity (categorical) were measured using the Fatigue Scale for Motor and Cognitive function (FSMC) as previously described (71). In brief, the assessment is a patient-reported outcome measure (PROM) with a generic tool that measures a variety of aspects of fatigue. It employs a Likert-type 5-point scale ranging from “does not apply at all” to “applies completely” producing a score between 1 and 5 for each scored question. For the general scale, the minimum value is 20 (no general fatigue at all) and the maximum value is 100 (very severe general fatigue). Besides distinguishing between physical and cognitive fatigue, this scale offers a subdivision into different grades of fatigue severity with a cut-off value of ≥63 considered as severe, ≥53–62 moderate and ≥43–52 mild general fatigue (71, 72).

Independent of care providers, diagnosis upon admission or current treatment, the standardized and validated questionnaires are completed by patients in most rehabilitation centers in Switzerland to ascertain perceptions of their fatigue status and severity, perceived level of general as well as physical and cognitive impairment.

##### Grip-strength

Hand grip strength in kg measured using a JAMAR™ hand dynamometer recorded as the average of three measurements of the dominant hand in the neutral position. All participants performed the test upon admission and at discharge at the occupational therapy department, in the morning, on the same dynamometer and supervised by the same assessors blinded to patient medication.

Prior research showed grip strength is inversely associated with cardiovascular and all-cause mortality events especially in individuals with T2D (73–77).

##### Peak-expiratory-flow-rate

Peak expiratory flow rate in L/minute registered as the best of 3 attempts using a non-digital vitalograph™ peak flow meter (Vitalograph GmbH Hamburg, Germany) was measured during the clinical visits by treating rehabilitation physicians as a measure of pulmonary function and expiratory muscle strength.

To produce accurate measurements, assessors provided explanations and demonstrated the measurement to the participants so that they could fully understand the procedure before the measurements were performed.

The peak expiratory flow rate, in the absence of obstructive lung disease, has been shown in previous studies to have a strong correlation with expiratory muscle strength which is equally closely associated to hand grip strength (78, 79). Furthermore, in older adults physical performance has been shown to be more strongly associated with respiratory muscle mass than total skeletal muscle mass (80).

##### Body-mass-index

Body mass index (BMI) in kg/m^2^ measured as weight (kg) divided by measured height (m) squared. Weight and height were measured upon admission by blinded certified nurses and physiotherapists, respectively, and entered into patient records. During stay, weight was controlled at regular intervals in the morning before breakfast during the clinical visits by blinded nurses.

BMI is a crude adiposity indicator with higher BMI being an independent predictor of higher mortality risk in the general population (76, 77). On the other hand, many large cohort studies of individuals with T2D and high BMI suggest higher BMI to be a protective factor for all-cause mortality, a phenomenon described as the “obesity paradox” (81–83). Consequently, in interventions inducing weight reduction in individuals with T2D, such as regular exercise training in rehabilitation in combination with diet and SGLT2 is, lifestyle changes focussed around improving physical performance are recommended to alleviate the potential risk of BMI reduction and cardiovascular mortality (84, 85).

##### Muscle-mass

**Figure 3:**
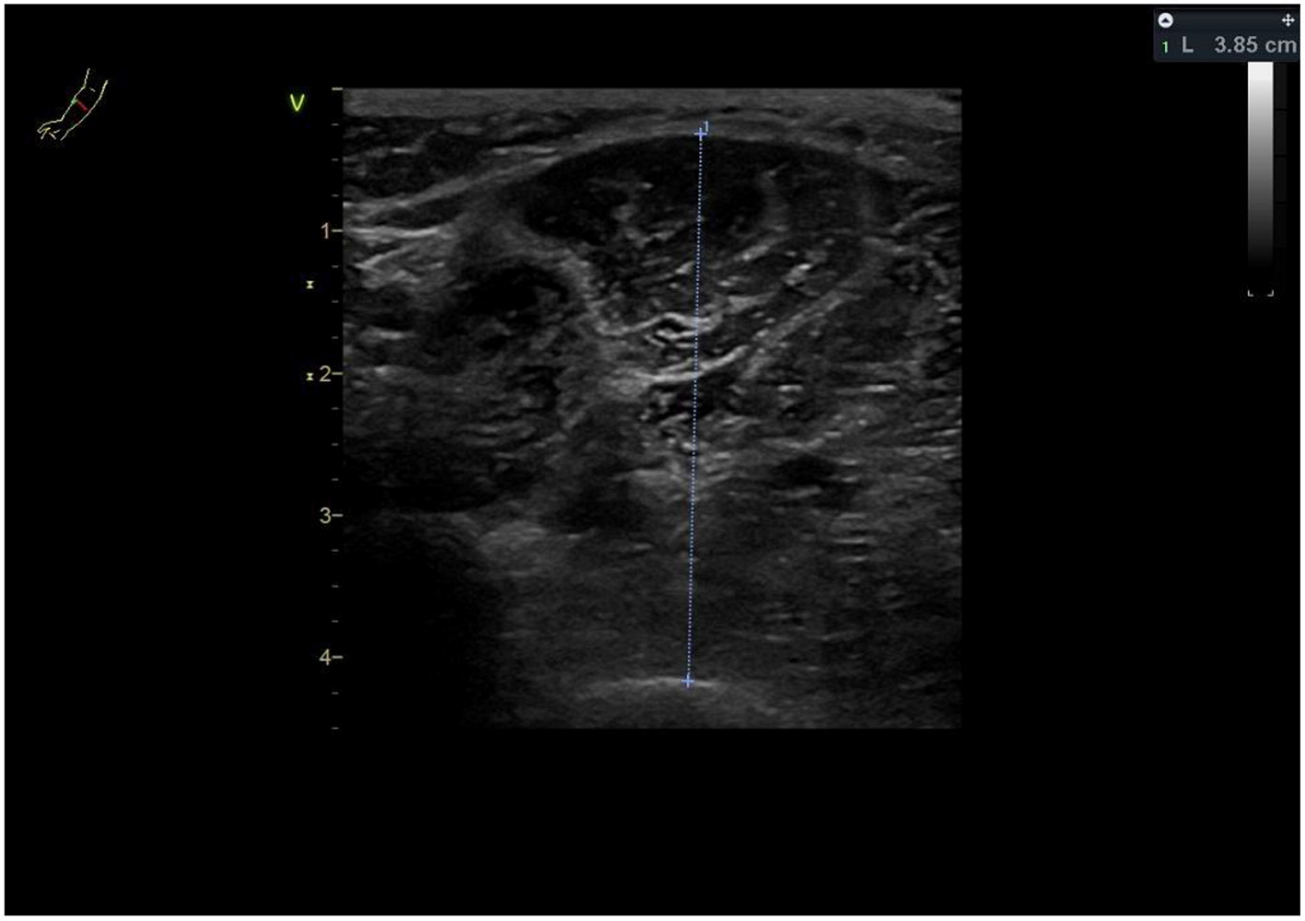
Muscle thickness ulna right forearm (here 3.85 cm).

Estimated appendicular skeletal muscle mass (ASM) in kg using the previously described Baumgartner equation [ASM = 0.2487 (weight) + 0.0483 (height)−0.1584 (hip circumference) + 0.0732 (Hand grip strength) +2.5843 (SEX) + 5.8828] (86), and muscle sonography [ASM = 4.89 × Muscle thickness-forearm (ulna) × Height (m)−9.15] (87–89). Hip circumference is measured during the clinical examination by treating rehabilitation physicians at the level of the widest protrusion of the buttocks with the patient standing erect and feet together. Muscle thickness of the forearm is measured at the anterior forearm at 30 % proximal between the styloid process and the head of the radius by a single trained and certified sonographer using a GE Vivid S70 N R2 (GE Medical Systems, Switzerland) with a 9L-D 2.4–10-MHz linear array transducer (Figure 3).

Not only is there a strong relation between muscle mass and physical performance (90), but also muscle mass as a measure of muscle quantity has been shown to have a strong correlation with ASCVD mortality with high muscle/low fat mass individuals having a lower risk of ASCVD and total mortality (91–93).

##### Muscle-quality

**Figure 4:**
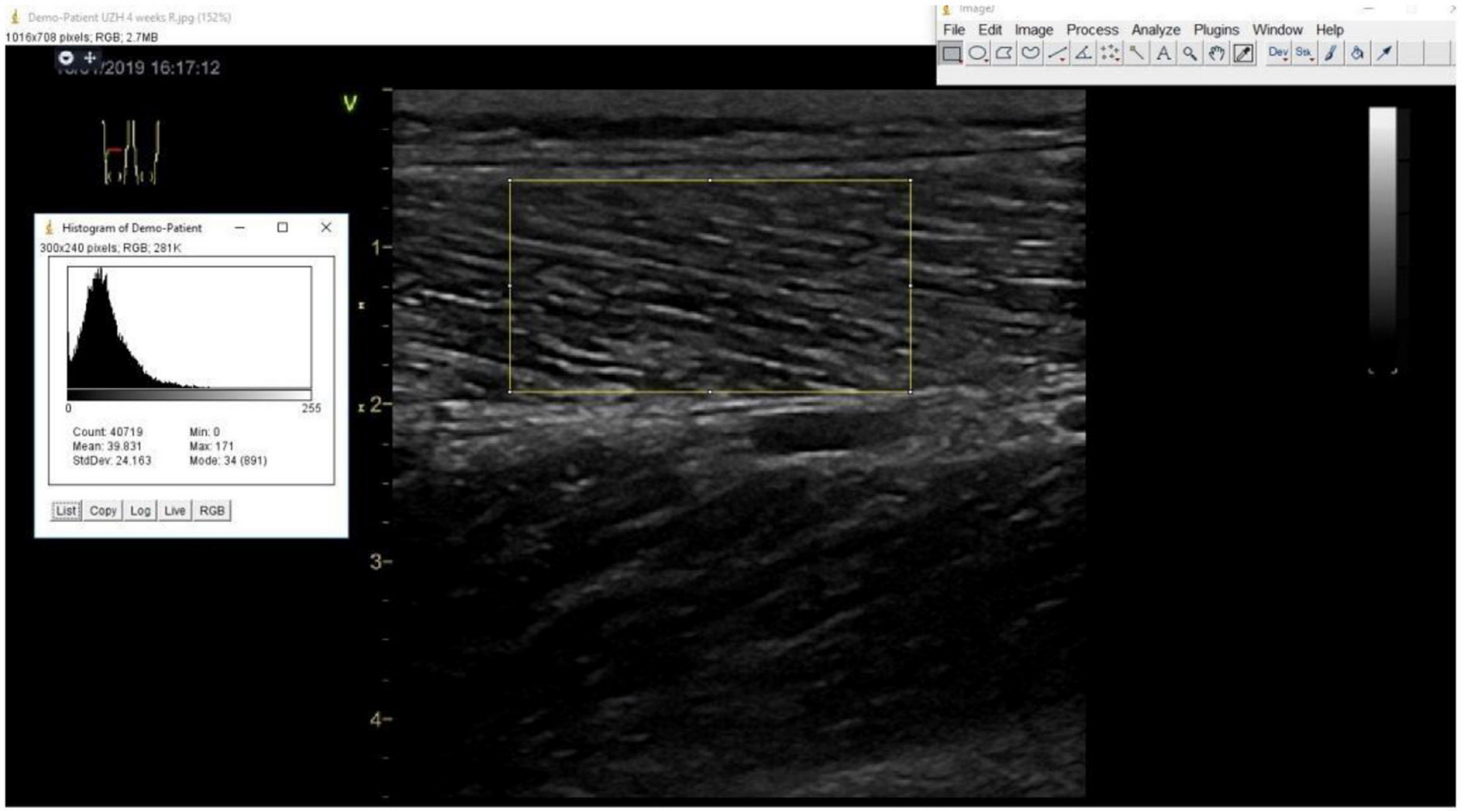
Measurement of echogenicity of the right vastus lateralis muscle using Image J software.

Muscle echogenicity of the vastus lateralis muscle on B-Mode ultrasound imaging obtained by a single trained and certified sonographer using a GE Vivid S70 N R2 (GE Medical Systems, Switzerland) with a 9L-D 2.4–10-MHz linear array transducer with duplicate assessment of outcomes using Image J software [Wayne Rasband, National Institute of Mental Health (NIH)] by an independent sonographer unaware of the treatment allocation following previously described protocols (94–96) as shown in Figure 4.

Muscle strength and physical performance are affected by not only muscle mass but also muscle quality which could be measured by echogenicity in ultrasound imaging (97, 98). Echogenicity reflects muscle quality, since non-contractile tissue associated with myosteatosis shows hyper-echogenicity (Figure 4) (98). Furthermore, individuals with T2D have been shown to have a an impaired microvascular blood flow in the muscles which could partially explain the impaired physical performance (99, 100).

To check for inter-rater and intra-rater reproducibility of the ultrasound measurements, especially when older individuals are examined, regular in-study reliability assessments (ICC) were conducted randomly on 30 patients of the PUSH study. For the ICC evaluation, measurements were repeated 3 times for each participant. The measured data shall be analyzed using a single-measurement, absolute-agreement, 2-way mixed-effects model and reported as ICC with 95% confident interval. Based on the ICC results, the measurements shall be classified according to the subgroups defined by Landis and Koch, in which ICC values below 0.00 are considered poor reliability, 0.00–0.20 slight reliability, 0.21–0.40 fair reliability, 0.41–0.60 moderate reliability, 0.61–0.80 substantial reliability, and 0.81–1.00 almost perfect reliability (101).

##### Functional-status

Functional independence measure (FIM™) as registered by blinded certified nursing staff. The FIM™ measures functional status, independent of diagnosis, using an 18-item, seven-level, ordinal scale intended to be sensitive to changes over the course of a comprehensive inpatient rehabilitation program (102).

The change in the FIM score has been shown to strongly correlate with long-term mortality in patients with CeVD admitted for rehabilitation (103).

##### Walking-aids

Use of walking aids (categorical) as documented by blinded physical therapists. As a measure of functional independence and balance, change in the need and use of walking aids is also a proxy measurement of change in physical performance in individuals with T2D (104, 105).

##### N-terminal-pro-brain-natriuretic-peptide

Fasting blood samples (serum or plasma as appropriate) are evaluated for circulating levels of N-terminal pro-brain natriuretic peptide (NT-pro-BNP) in ng/L [1 Nanogram per liter (ng/l) = 1 Picogram per milliliter (pg/ml)] measured by laboratory technicians unaware of study group allocation.

In patients with ASCVD, NT-pro-BNP levels have been shown to predict physical performance and cardiovascular events (106–109).

##### Anthropometric-indices

Anthropometry comprising of mean waist and hip circumferences in cm, waist-to-height, and waist-to-hip ratios. Waist circumference was measured during the clinical examination by treating rehabilitation physicians at the level of the umbilicus with the patient standing erect and feet together.Waist circumference has been demonstrated to be a modifiable factor associated with post-rehabilitation physical performance and function (110). Additionally, both waist-to-height and waist-to-hip ratios have been associated with cardiovascular events (111–113).

##### Parameters-of-muscle-physiology

Fasting blood samples (serum or plasma as appropriate) evaluated for biomarkers of muscle response to training including creatinine kinase (CK) in U/L, lactate dehydrogenase (LDH) in U/L (114), and biomarkers likely influencing both musculoskeletal function and cardiovascular disease including serum magnesium (115, 116) in mmol/L, serum ionized calcium and serum vitamin D levels (117).

##### Other-biomarkers-associated-with-fatigue

Fasting blood samples (serum or plasma as appropriate) were evaluated for biomarkers of iron deficiency including serum ferritin in μg/L, transferrin saturation (TSAT) in % (118).

#### Safety outcomes

The safety outcomes included adverse event (AE) reporting, hemoglobin, liver and kidney function, electrolytes, dehydration, polyuria, nocturia, urogenital infections, (euglycemic) diabetic ketoacidosis. A general physical examination was performed at baseline and at the end of the study. Patients were specifically asked at the regular clinical visits about AEs. Each AE was registered in a standardized manner with laboratory findings in the study registry. In addition, at baseline and at week 4, calculated BUN creatinine ratio and ultrasound measurements of the inferior vena cava were employed as surrogate markers of dehydration (119, 120).

### Sample size and data analysis

#### Sample size

Based on the results of the cardiovascular heart study of Enright et al., among 2,281 community dwelling adults ≥68 years old, the expected mean 6-min walk distance (6MWD) was 344 ± 88 m which is the set target of exercise therapy in rehabilitation (45). Centered on our clinical observations and on the results of a small pilot study in the clinical rehabilitation of patients with cerebrovascular disease (121), we assumed a mean 6-MWD at baseline of 250 m with an anticipated improvement of 80 ± 40 m after 4 weeks of exercise therapy. To detect an additional difference of ±15%, as reported in previous studies on this subject (28), within the group as well as between any 2 groups with power of 80 % and an alpha error of 5% using a 2-sided test after 4 weeks, the sample size required was 130 participants per group. Based on our admission statistics, the attrition rate for administrative reasons, mainly being lack of insurance coverage for a treatment duration of at least 20 days, was estimated at 15 %, while 85 % of participants were expected to complete the week 4 follow-up. To this end, 150 participants per group needed to be enrolled (450 participants in total).

#### Data analyses

Demographic data will be examined using independent-samples *t*-tests and Fisher's exact tests for dichotomous variables. Independent-samples *t*-tests and repeated-measures ANOVAs will be employed to compare changes in physical performance after 4 weeks. Differences between primary and secondary outcome variables after 4 weeks will also analyzed by linear mixed-effects models (LMMs). The time factor for the model will be set at baseline and week 4. The models will include model-based adjustments for baseline characteristics influencing physical performance in such cohorts: (1) age, sex, BMI, waist circumference, diabetes duration and history of cardiovascular disease.

To minimize the bias that could result from participants in the 3 groups having highly variable duration of stay, the prospective analyses will be performed on a modified intention-to-treat and per protocol basis. The modified intention-to-treat population will be defined as participants who have been categorized to a study group and have at least one follow-up measurement of the primary outcomes, regardless of treatment adherence or duration of stay. The “last observation carry-forward method” will be used in the absence of complete follow-up data. For “per protocol” analysis, only those participants who have follow-up measurements after a minimum treatment duration or length of stay of ≥20 days will be included.

Sensitivity analyses will be performed to account for potentially non-ignorable missing data due to dropout as well as the possible impact of other potentially influential variables. A Bonferroni correction will be applied to the LMM analyses of the primary and secondary outcomes.

Pre-specified subgroup analyses will be conducted; these will include subgroups defined according to the various entities of ASCVD, gender, age groups, and other factors.

## Discussion

The PUSH is a a real-world, 4-week prospective, three-arm observational study designed to evaluate the impact of glycemic control and guideline-directed medical therapy with an SGLT2i on physical performance of patients with established ASCVD or high cardiovascular risk.

Prior research suggests that there is a strong correlation between physical performance and the risks of cardiovascular disease and mortality (62, 122). It is also clear that treatment with SGLT2is reduces the risks of heart failure hospitalisations, progression of renal disease, cardiovascular disease and mortality in individuals with T2D with changes seen early within a few weeks after the begin of treatment (20–22). Therefore, unlike previous pilot studies on the subject (27–31), the PUSH incorporated strategies to investigate change in physical performance in a larger cohort of individuals with established ASCVD or high cardiovascular risk under guideline-directed medical therapy. Because change in physical performance is highly dependent on both oxygen delivery and utilization systems (123), the PUSH assesses cardiac, respiratory and muscle parameters related to physical performance. Thus, much effort was made in the design to have an extensive assessment of variables impacting exercise capacity as previously described in similar cohorts (1). In analyzing or adjusting for these variables, the results of the PUSH could be of clinical relevance in the management of patients with T2D and established ASCVD or high cardiovascular risk.

Due to demographic factors, the clinical and economic costs related to ASCVD are expected to increase dramatically in coming years. Consequently, the identification of therapy regimes capable of reducing ASCVD disease burden is an important goal with remarkable public health repercussions (124).

The PUSH design however has some limitations. First, the non-randomized design precludes analyses and conclusions of the causal relationships among variables. Second, despite efforts to address the internal validity threats, the selection bias of using glycemic control to determine participants for the guideline-directed medical therapy with an SGLT2i remains an important threat in the interpretation of the results even though a previous study in this field did not show any negative impact of suboptimal glycemic control under treatment with an SGLT2i (dapagliflozin 10 mg) on the physiological adaptation to endurance exercise training (31). Accordingly, studies on changes in physical performance under SGLT2is in patients with both inadequately and adequately controlled T2D are still warranted. Third, the prospective design of this study with a follow-up of just 4 weeks addresses solely the hypothesis of early changes in physical performance under guideline-directed medical therapy with an SGLT2i. Further studies will be necessary to investigate, if present, the extent and sustainability of these early changes over a longer period of observation with or without endurance exercise therapy. Finally, we will not compare participants to those who did not receive inpatient rehabilitation after a medical or surgical condition warranting inpatient rehabilitation, as such, the potentiating effect of exercise therapy during rehabilitation on the results shall not be easily quantified.

## Study status

The first participant was recruited in January 2018. Data collection was concluded in September 2020 when the target of 450 participants was met. The SARS-COVID global pandemic delayed patient enrollment and data collection between February and September 2020 but neither relevantly influenced the study protocol nor the rehabilitation program. The publication of the results is expected in 2022.

## Ethics Statement

Written informed consent was obtained from the individual(s) for the publication of any potentially identifiable images or data included in this article.

## Author Contributions

DF: project administration, conceptualization, methodology, investigation, visualization, and writing original draft. EK: conceptualization, resources, validation, data curation, visualization, and writing review and editing. LP: resources provision of study materials and patients. MHo: resources, visualization, and writing review and editing. MHu and EL: resources, validation, data curation, and visualization. YF: conceptualization, methodology, and formal analysis. AB and TM: formal analysis and writing review and editing. MW: writing review and editing and supervision. All authors contributed to the article and approved the submitted version.

## Funding

The PUSH was approved and funded by the Berner Klinik Foundation, Bern, Switzerland. The foundation is a non-profit organization focused on clinical rehabilitation. External funding was neither sought nor endured from previous research projects in the institution. The investigators designed and conducted the study, will perform all study analyses and have contributed and are responsible for the contents of this manuscript. The principal investigator will have full access to all the data and the final responsibility for the decision to submit for publication.

## Conflict of interest

The authors declare that the research was conducted in the absence of any commercial or financial relationships that could be construed as a potential conflict of interest.

## Publisher's Note

All claims expressed in this article are solely those of the authors and do not necessarily represent those of their affiliated organizations, or those of the publisher, the editors and the reviewers. Any product that may be evaluated in this article, or claim that may be made by its manufacturer, is not guaranteed or endorsed by the publisher.
